# Feasibility of Using an Artificial Intelligence-based Telephone Application for Dietary Assessment and Nudging to Improve the Quality of Food Choices of Female Adolescents in Vietnam: Evidence from a Randomized Pilot Study

**DOI:** 10.1016/j.cdnut.2023.102063

**Published:** 2023-12-13

**Authors:** Bianca C Braga, Phuong H Nguyen, Lan Mai Tran, Nga Thu Hoang, Boateng Bannerman, Frank Doyle, Gloria Folson, Rohit Gangupantulu, Naureen Karachiwalla, Bastien Kolt, Peter McCloskey, Giordano Palloni, Trang Huyen Thi Tran, Duong Thuy Thi Trơưng, David Hughes, Aulo Gelli

**Affiliations:** 1 Friedman School of Nutrition Policy and Science, Tufts University, Boston, MA, United States; 2 Nutrition, Health and Diet, International Food Policy Research Institute, Washington, DC, United States; 3 Thai Nguyen University of Pharmacy and Medicine, Thai Nguyen, Vietnam; 4 Hubert Department of Global Health, Rollins School of Public Health, Emory University, Atlanta, GA, United States; 5 National Institute of Nutrition, Hanoi, Vietnam; 6 Noguchi Memorial Institute for Medical Research, College of Health Sciences, University of Ghana, Legon, Ghana; 7 College of Agricultural Sciences, Pennsylvania State University, State College, PA, United States

**Keywords:** adolescence, dietary assessment, dietary intake, dietary quality, feasibility, randomized controlled trial, artificial intelligence, telephone application, e-health, m-health

## Abstract

**Background:**

Adolescent nutrition has faced a policy neglect, partly owing to the gaps in dietary intake data for this age group. The Food Recognition Assistance and Nudging Insights (FRANI) is a smartphone application validated for dietary assessment and to influence users toward healthy food choices.

**Objectives:**

This study aimed to assess the feasibility (adherence, acceptability, and usability) of FRANI and its effects on food choices and diet quality in female adolescents in Vietnam.

**Methods:**

Adolescents (*N* = 36) were randomly selected from a public school and allocated into 2 groups. The control group received smartphones with a version of FRANI limited to dietary assessment, whereas the intervention received smartphones with gamified FRANI. After the first 4 wk, both groups used gamified FRANI for further 2 wk. The primary outcome was the feasibility of using FRANI as measured by adherence (the proportion of completed food records), acceptability and usability (the proportion of participants who considered FRANI acceptable and usable according to answers of a Likert questionnaire). Secondary outcomes included the percentage of meals recorded, the Minimum Dietary Diversity for Women (MDDW) and the Eat-Lancet Diet Score (ELDS). Dietary diversity is important for dietary quality, and sustainable healthy diets are important to reduce carbon emissions. Poisson regression models were used to estimate the effect of gamified FRANI on the MDDW and ELDS.

**Results:**

Adherence to the application was 82% and the percentage of meals recorded was 97%. Acceptability and usability were 97%. MDDW in the intervention group was 1.07 points (95% CI: 0.98, 1.18; *P* = 0.13) greater than that in the control (constant = 4.68); however, the difference was not statistically significant. Moreover, ELDS in the intervention was 1.09 (95% CI: 1.01, 1.18; *P* = 0.03) points greater than in the control (constant = 3.67).

**Conclusions:**

FRANI was feasible and may be effective to influence users toward healthy food choices. Research is needed for FRANI in different contexts and at scale.

The trial was registered at the International Standard Randomized Controlled Trial Number as ISRCTN 10681553.

## Introduction

Nutrition during adolescence is critical for health and development [[1], [2], [3]]. Adolescence is also a pivotal life stage for forming eating habits [4,5], which can carry through adulthood and have long-term health effects, including for the offspring of females [6,7]. However, adolescent nutrition has faced a pervasive policy neglect globally, partly because of data gaps on food consumption and diets [8,9]. Dietary assessment is complex and costly [10], but electronic-based dietary assessment is rapidly growing with the sheer number of people who own cell telephones and use telephone applications worldwide [11]. Researchers and participants may be more inclined toward electronic than conventional dietary assessment methods owing to the perceived gains in precision and in the time saved in the assessment [12,13]. The automation and standardization of real-time food consumption data collection and analysis with mobile telephones have the potential to reduce research burden and cost [14]. Smartphone has enormous potential as instrument of data collection [15], while use of smartphone-based interventions may improve the quality of food choice [16,17]. Despite these advantages, electronic-based assessments are often used in dietary surveys and interventions without previous validity and usability assessments [18].

The Food Recognition Assistance and Nudging Insights (FRANI) application was developed to be used by adolescents in Vietnam and Ghana [19,20]. FRANI validation studies provided reliable estimation of nutrient consumption of female adolescents compared with 24-h recalls and weigh food records [[21], [22], [23]]. This novel form of data collection uses artificial intelligence to recognize foods in photographs recorded by study participants and integrates this with information on food recipes and composition to accurately estimate food and nutrient intake [[21], [22], [23], [24]]. FRANI’s user interface is gamified, or uses game elements, such as point scores, badges, progress bars, leaderboards, performance graphs, social influence, and interaction, to promote nutrition learning and healthy eating [[25], [26], [27]]. Individuals use social norms to adapt personal behavior [28]. Important evidence gaps exist on the effectiveness of introducing game elements to improve nutrition behavior, but emerging evidence suggests it can be an effective strategy [29,30]. Gamification has encouraged fruit and vegetable consumption [31] and has helped to improve nutrition knowledge [30,32] and to change the nutrition quality of food purchases in online grocery shopping [33,34]. This study assessed the feasibility (as captured through adherence, acceptability, and usability) and effects of using FRANI to improve the quality of food choice and diet of female adolescents in Vietnam.

## Methods

### Study design and participants

**FIGURE 1:**
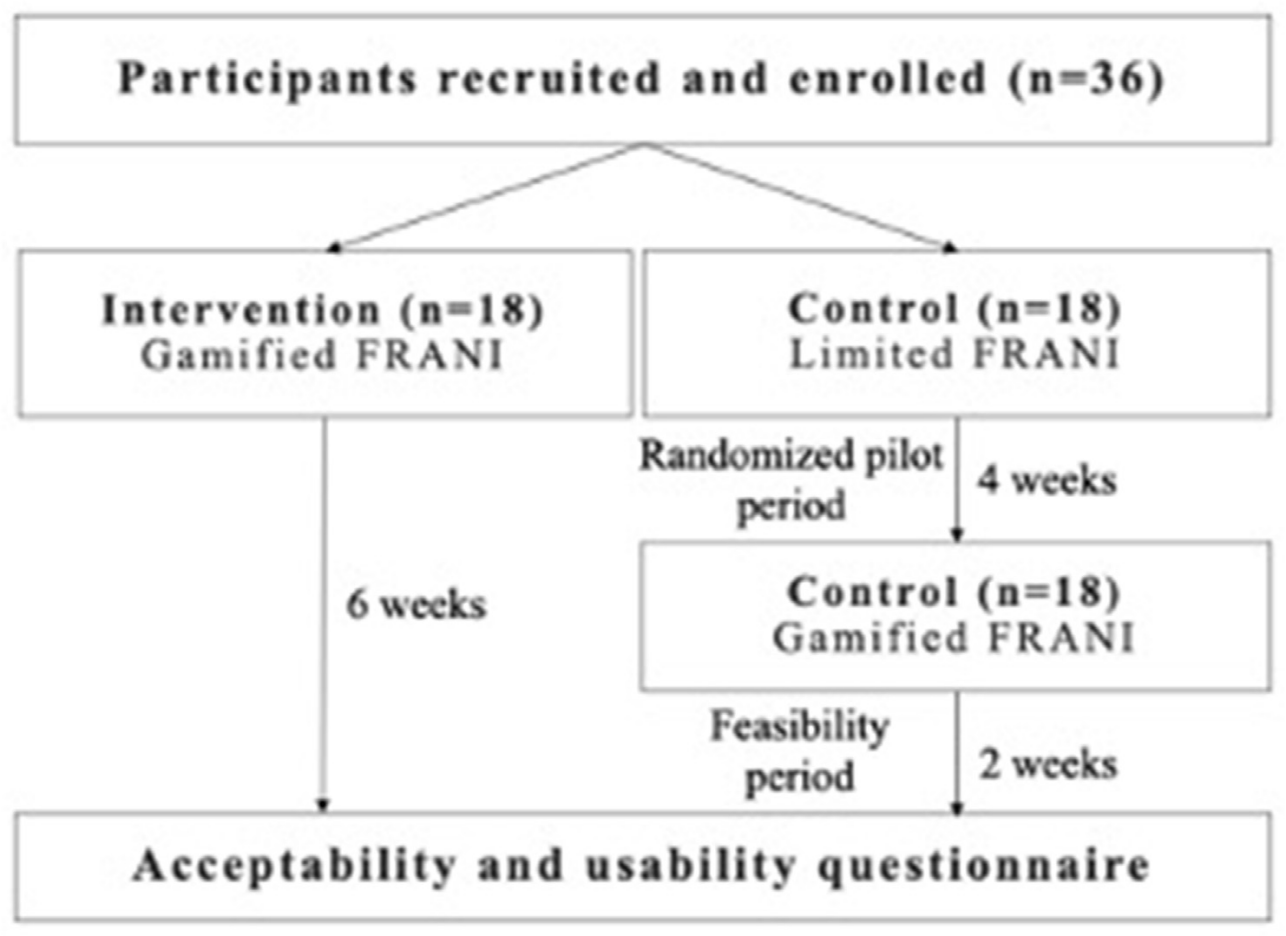
CONSORT diagram. All participants recruited to the study were enrolled.

**FIGURE 2:**
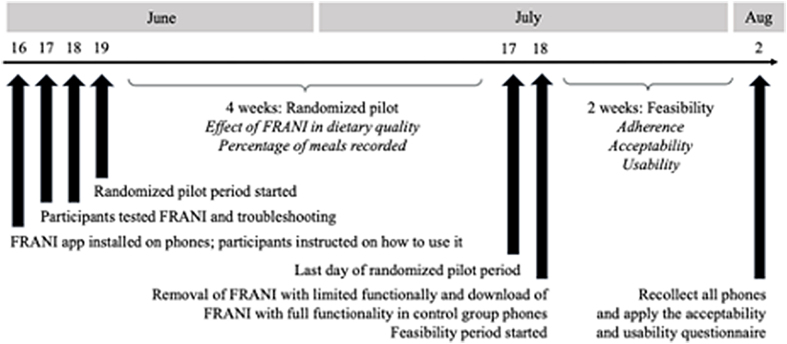
Timeline of the randomized pilot and feasibility study.

This 6-wk study had the following 2 parts: *1*) a randomized controlled pilot, conducted in the first 4 wk, and *2)* a feasibility study, conducted in the last 2 wk (Figure 1). Thirty-six female adolescents were randomly selected through a lottery from a selected public school in Thai Nguyen, a secondary city in Vietnam. The school was chosen based on proximity and existing relationships with the research team at the Thai Nguyen National Hospital. Adolescents were eligible for inclusion if they met the following criteria: female, aged between 12 and 18 y, capable of using the smartphones with FRANI application (provided by the project), and willingness to use it for 6 wk. Recruitment was conducted by visiting identified adolescents at their school a week before the intended actual research day. Field enumerators met with adolescents and send the study document to caregivers, presented study purpose and procedures, sought informed consent by parents and assent by adolescents, and made appointments for the data collection week. Before the pilot study start, participants were randomly allocated into intervention and control groups. Participants in the control group received telephones with a version of FRANI with functionality limited to dietary assessment (here referred to as FRANI control). The intervention group received telephones with a gamified FRANI interface. Participants in both groups were separately trained by the research team on how to record food consumption on FRANI (Figure 2). A 2-d troubleshooting period followed, when participants practiced using FRANI to record food consumption and problems related to application use were resolved. On day 4, participants in both groups were free to use FRANI for the following 4 wk. The feasibility study started in the fifth week, on completion of the randomized pilot period. The participants in the control group had FRANI control removed and the gamified FRANI version installed on their telephones. Both intervention and control groups were instructed to use the gamified FRANI for 2 additional weeks. There were in-person weekly sessions with intervention and control FRANI groups separately to check and motivate the collection of data and troubleshoot problems in case there was any.

### FRANI functionality description

#### Background information used to develop FRANI.

Qualitative research undertaken as part of this project found that female adolescents from Vietnam had previously tried to track food consumption using commercial applications but stopped because they were difficult to use and time intensive [19,20]. Uploading food consumption generally involved a multistep process that included finding each food or ingredient separately on a dropdown menu and guessing their weight [20]. Adolescents believed their diet was unhealthy but did not trust the nutrition advice from those applications and followed fad diets [19,20]. Moreover, it has been shown that commercial applications might evoke unhealthy behavior [35]. Recent findings suggest that effective strategies to improve adolescent health must be multifaceted and adapted to local context [9]. FRANI was designed to be a practical dietary assessment tool and promote healthy eating based on the behavioral scientific literature and the food-based Dietary Guidelines of Vietnam [19,20,36]. The interface was designed considering challenges mentioned in focus-group discussions with potential users, including the impossibility of separating individual portions from shared family meals (because this would be considered culturally inappropriate), and confusion about what source of nutrition information should be trusted [20].

#### Difference between the gamified FRANI and FRANI control versions.

The differences between the gamified and control versions are summarized in the Supplemental Table 1. In brief, the gamified FRANI and FRANI control applications were similar in account registration and login, meal recording, and notifications to remind participants to record meals. FRANI gamified and control applications had slide bars to indicate the proportion consumed of meals recorded when food was shared with family or friends. The gamified version has some additional functions that the limited version does not have, such as setting goals, scores and statistics, and daily report. In this article, we use the word “meals” to refer to both meals and snacks, but both versions of FRANI had a more comprehensive term (such as *bữa ăn, thức ăn*, and *đồ ăn nhẹ*) in Vietnamese to communicate the concepts of meals and snacks to participants. Meals could be recorded manually, by taking photographs, or a combination of both; the information was automatically saved. Taking pictures to record meals was more practical and time saving than manual entry. But, if a whole meal or part of it was not recognized from a picture by FRANI, the participant could input the information manually by looking for the food in a dropdown menu. Only participants using the gamified version of FRANI could set food consumption goals. Food consumption goals were goals related to the consumption of food groups from the Minimum Dietary Diversity for Women (MDDW) and the Eat-Lancet Diet Score (EDLS) in the course of 1 d that were point scored and summarized in the statistics page daily and over the latest 7-d period. The Outcome Measure section has more details about the food consumption scores. Both study groups received a final prompt every evening to log foods consumed that had not been recorded yet to avoid incomplete food records and include the number of meals that had not been recorded to classify that food record as complete or incomplete. Only the gamified FRANI sent daily reports in the evenings summarizing food consumption scores.

#### Gamified FRANI prompts to change behavior.

**FIGURE 3:**
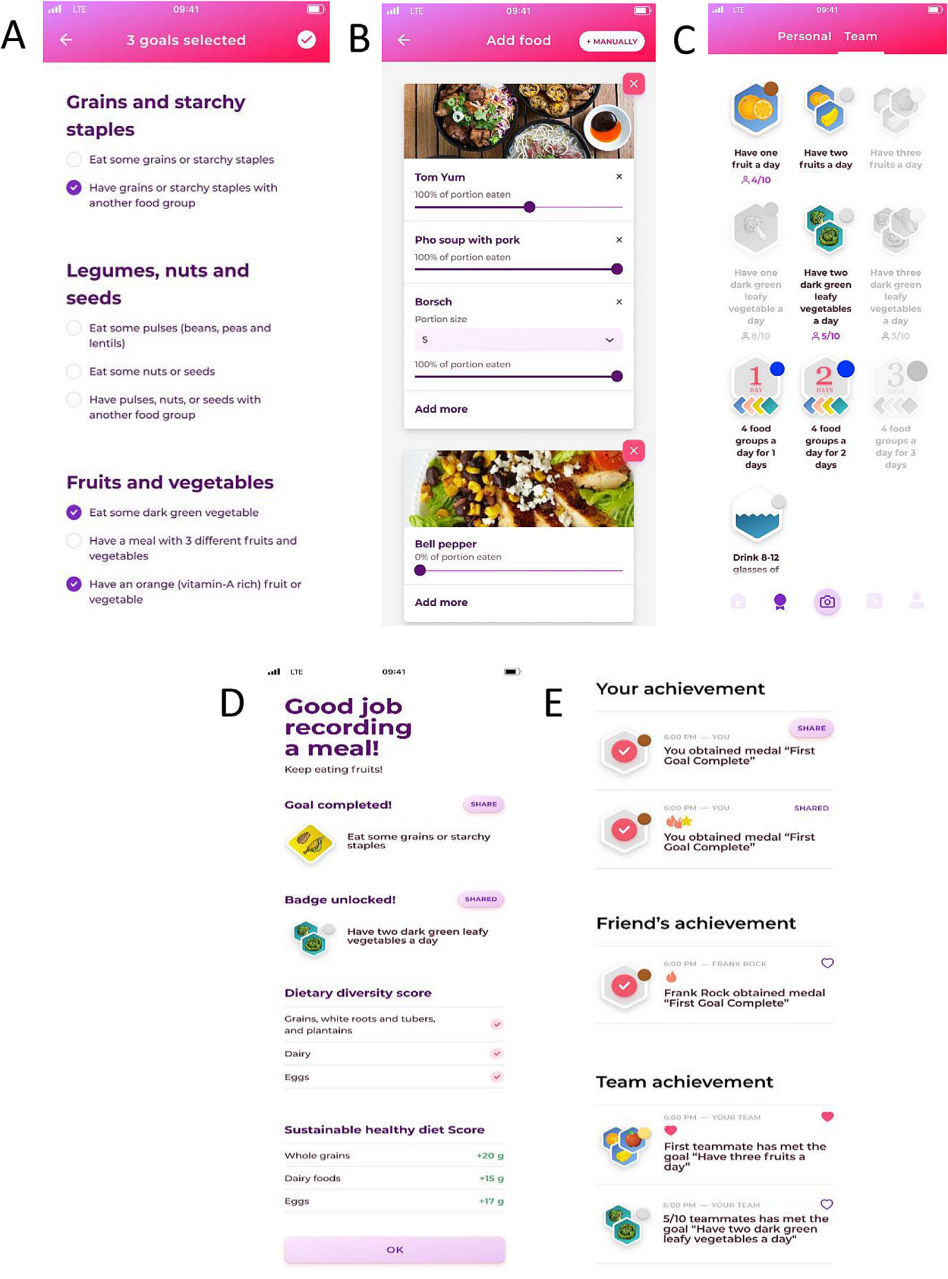
Wireframe A shows options of food consumption goals for 3 of the 5 food groups (in addition to dairy and meat). Wireframe B shows the slide bars to indicate the proportion consumed of the meal recorded, and wireframe C shows the badges. Wireframe D is the home dashboard, and wireframe E is the activity screen, where users can share achievements with friends, which can react to it by clicking in the hearts. Participants used FRANI in Vietnamese.

The gamified FRANI application automatically tracked progress against personal food group–based goals and awarded badges as incentives for achieving individual-based or team-based goals. Participants could join teams for 1 wk to have shared goals with other team members (“Eat vegetables every day for 1 week” and/or “Eat fruits every day for 1 week” and/or “Drink 6 glasses of water every day for a week”). For example, if the team-based goal was to eat fruits every day, the team members would be rewarded with a badge in case all team members consumed fruits that day. The point scores for each team member would have increased as well. Participants could share their badge achievements in the activity screen and receive positive feedback (eg, hearts, stars, or likes) from other users, as in social media. Each food group had its own badge design, and participants were leveled up from bronze to silver and gold depending on how many days in a row they achieved dietary goals. The gamified FRANI application also has badges for dietary diversity. The food consumption goals were based on the food groups of the My Plate application [37] and adapted to the Vietnamese context according to the food-based dietary guideline of Vietnam and information from previous focus-group discussions [20,36]. Examples of the gamified FRANI user interface (translated into English) are shown in Figure 3 and Supplemental Figure 1.

### Outcome measures

The primary outcome of the study was the feasibility of using FRANI, as measured by adherence, acceptability, and usability during the feasibility study period (the last 2 wk of the study), when participants from both groups used the gamified FRANI application. Adherence was defined as the number of completed daily food records divided by the total number of food records. Food records were considered complete if all meals consumed in a given day were recorded. Food records were considered incomplete if 1 meal or more was not recorded. FRANI asked participants to self-report at the end of each day the number of meals they did not record throughout that day. A target of ≥70% was defined a priori as necessary for the group of participants to be considered adherent based on findings from a literature review [19]. Acceptability and usability were assessed using a questionnaire based on a Likert scale from 1 (strongly agree) to 5 (strongly disagree) at the end of the feasibility period. We based the questions on the information provided by the participants themselves in 4 focus-group discussions [19,20]. They talked about how much they liked the first version of the application, the badges, the daily and weekly diet reports, the statistics, the team goals, how much they believed the first version would be useful to help them to eat healthily [19,20]. Because these were the topics that Vietnamese adolescents talked about the most, we decided to have acceptability and usability questionnaire based on the same topics. Acceptability related to issues of likeability and satisfaction, whereas usability related to use and intent to continue using FRANI beyond the study period. FRANI was considered acceptable or usable if the sum of points was 30 or less on a scale from 10 to 50 points.

The secondary outcomes of the study were the percentage of meals recorded, and dietary quality in the first 4 wk of the study. The percentage of meals recorded was the number of meals recorded divided by the number of meals consumed. Dietary quality was measured by the following 2 indicators: *1*) the MDDW, a measure validated against micronutrient adequacy for women at reproductive age [[38], [39], [40]]; *2*) the ELDS, a measure to promote human and environmental health, validated for women at reproductive age in Vietnam [[41], [42], [43]]. The daily MDDW score went from 0 to 10 (consumption of 0–10 MDDW food groups), and the daily ELDS went from 0 to 14 (consumption of 0–14 Eat-Lancet Diet food groups) [39,41]. For ease of participant’s interpretation, these indicators were labeled on FRANI as the Dietary Diversity Score and Sustainable Healthy Diet Score, respectively.

In addition, we included the Global Diet Quality Score (GDQS) because it is the only indicator so far validated for dietary quality outcomes related to both nutrient adequacy and noncommunicable disease for women of reproductive age globally [15]. The GDQS was included only as exploratory analysis because this indicator was published only after this study was preregistered. The GDQS ranges from 0 to 49 points according to the quantity consumed of 25 food group (16 healthy, 7 unhealthy, and 2 unhealthy when consumed excessively) [15]. During the validation phase of the project, 2 researchers classified the foods recognized by FRANI into the food groups of the MDDW, the ELDS, and the GDQS, one by one [15,39,41]. The ELDS and the GDQS account for the weight eaten of a certain food to classify it into consumed or not consumed within the healthy level. If the participant ate, for example, ≤28 g of meat on a certain day, she would have received 1 for meat consumption in the ELDS [41]. The MDDW does not account for the weight eaten of a certain food to classify it into consumed or not consumed [39]. FRANI was programmed to recognize food volume and calculate weight based on the Vietnamese Food Composition Table [44]. During the pilot study, FRANI automatically classified foods recognized from pictures into the MDDW, ELDS, and GDQS food groups based on the previous classification by the researchers. Then, FRANI automatically scored daily food group consumption. A researcher double-checked FRANI’s food weight and daily scores automation results by converting the volumes of foods into weight and calculating the daily scores again. FRANI automation correctly recognized the foods from pictures, correctly attributed them to the food groups, and correctly calculated the daily scores. The food consumption scores could only be seen by the gamified FRANI users.

### Data analysis

**FIGURE 4:**
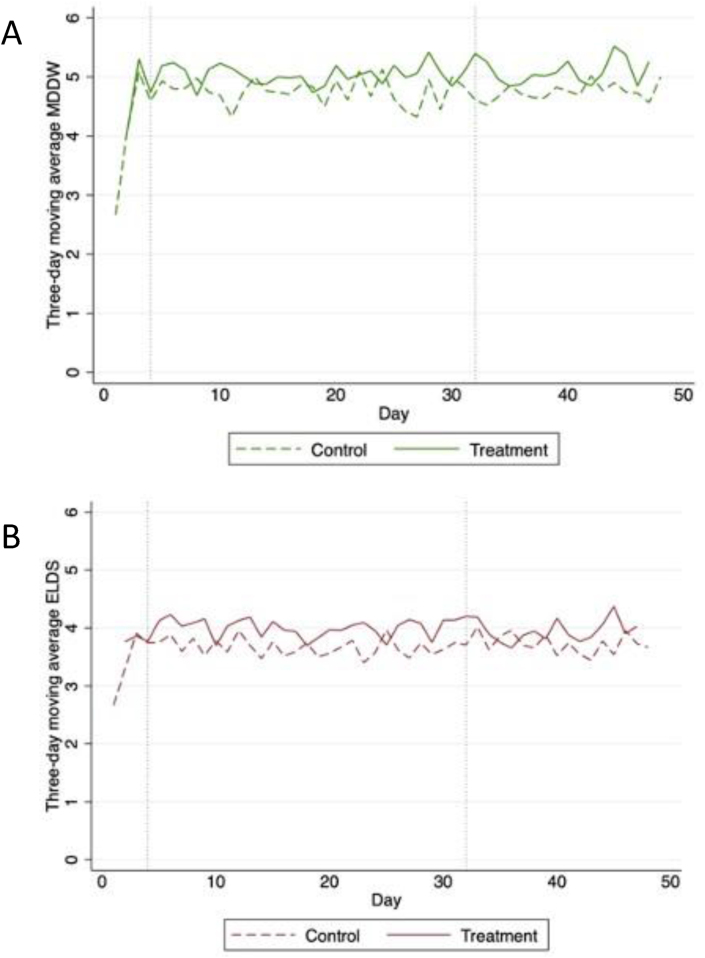
Centered 3-d moving average of Minimum Dietary Diversity for Women score (A) and Eat-Lancet Diet Score (B) was used to smooth noise in the data. From left to right, horizontal reference lines mark the start of the randomized pilot period, and the end of randomized periods and start of the feasibility period. Solid lines are for the intervention group and dashed for the control group. The figure shows that dietary diversity among the treatment group is higher than that of the control group independently of the score used.

Descriptive statistics summarized parent and household socioeconomic characteristics and adolescents’ smartphone use for intervention and control groups. The effect of the gamified FRANI application on dietary quality was measured by the difference between the MDDW and the ELDS between participants using the gamified and control FRANI (Figure 4). This was estimated through the analysis of food consumption data from the randomized pilot period (first 4 wk of the study), when participants were individually randomly assigned to use one of the 2 different versions of FRANI. Poisson regression models included random effects at individual level to account for the repeated measures. The bivariate regression had intervention (use of gamified FRANI) as the only independent variable, whereas the multivariate regression added time and the interaction of time and intervention. Description of best-fit multilevel models for health interventions that use gamification have a time-variant predictor [45]. The interaction between the time variable and the dummy for FRANI version was included because users take time to adapt to new technologies, so the effect of FRANI could change with time. Additional exploratory analysis was undertaken to estimate the effects of the intervention on the probability of consuming each food group of the MDDW and of the ELDS using logistic regressions. The data analyses were performed using intention-to-treat approach, and a *P* value of <0.05 was considered statistically significant.

### Ethical approval

The study was approved by the institutional review board of the International Food Policy Research Institute on 29 April 2020 (registration number 00007490) and the Thai Nguyen National Hospital on 14 April 2020 (protocol code 274/ĐĐĐ-BVTWTN). The study was registered under the International Standard Randomized Controlled Trial Number on 12 November 2021 (ISRCTN 10681553), and the protocol of the study was published on 6 December 2022, in *Frontiers in Digital Health* [46]. All procedures were undertaken in accordance with the tenets of Declaration of Helsinki.

## Results

### Baseline characteristics

Comparison of baseline demographic and socioeconomic characteristics suggests that participants in both groups were similar at the start of the study period for all variables but number of people who lived in the household (*P* = 0.026) (Table 1). For example, the number of household assets, such as TV (*P* = 0.349), computer (*P* = 0.710), refrigerator/freezer (*P* = 0.460), and air conditioner (*P* = 0.491) were similar between groups, so as the frequency and reasons to use smartphone for calls (*P* = 0.641), message (*P* = 0.560), and listen to music (*P* = 0.154). The level of education and type of occupation of parents also did not differ between the groups.

**TABLE 1:** Characteristics of the pilot study participants, their parents, and households (*N* = 36).

Characteristics of the household and participants	Control (%)	Intervention (%)	*P*	Characteristics of the parents	Control (%)	Intervention (%)	*P*
Household size, mean (SD)	5.1 (1.5)	4.2 (0.9)	0.026^1^	Mother’s education, *n* (%)			0.264
Household assets				Less than high school	4 (22.2)	4 (22.2)	
TV	17 (94.4)	18 (100)	0.349	High school	12 (66.7)	8 (44.4)	
Computer	10 (55.6)	13 (72.2)	0.710	College	2 (11.1)	5 (27.8)	
Refrigerator/freezer	18 (100)	18 (100)	0.460	Postgraduate	0 (0)	1 (5.6)	
Air conditioners	11 (61.1)	10 (55.6)	0.491	Father’s education, *n* (%)			0.756
Washing machine	16 (88.9)	15 (83.3)	0.217	Less than high school	7 (38.9)	10 (58.9)	
Gas cooker/stove	18 (100)	18 (100)	0.623	High school	10 (55.6)	5 (29.4)	
Bicycle	11 (61.1)	14 (77.8)	0.112	College	1 (5.6)	1 (5.9)	
Electric bicycle	7 (38.9)	8 (44.4)	0.558	Postgraduate	0 (0.0)	1 (5.9)	
Motorcycle	18 (100)	18 (100)	0.043^1^	Mother’s occupation			0.594
Water heater	17 (94.4)	17 (94.4)	0.710	Farmer	2 (11.1)	6 (33.3)	
Car	5 (27.7)	1 (5.6)	0.191	Blue-collar worker	5 (27.8)	2 (11.1)	
Uses smartphone	15 (83.3)	16 (88.9)	0.764	White-collar worker	1 (5.6)	2 (11.1)	
Uses smartphone 5–7 times/wk	17 (94.4)	18 (100)	0.324	Unskilled worker	9 (50.0)	5 (27.8)	
Reason to use telephone				Stay-at-home parent	0 (0)	1 (5.6)	
Call	16 (88.9)	15 (83.3)	0.641	Other	1 (5.6)	2 (11.1)	
Messaging	17 (94.4)	16 (88.9)	0.560	Father’s occupation			0.566
Listen to music	18 (100)	18 (100)	0.154	Farmer	2 (11.1)	4 (23.5)	
Movie	18 (100)	18 (100)	0.324	Blue-collar worker	4 (22.2)	4 (23.5)	
Game	23 (63.9)	12 (66.7)	0.738	White-collar worker	2 (11.1)	2 (11.8)	
Social media	18 (100)	18 (100)		Unskilled worker	8 (44.4)	6 (35.3)	
Internet (news)	18 (100)	16 (88.9)	0.154	Stay-at-home parent	0 (0)	0 (0)	
For learning	18 (100)	15 (83.3)	0.074	Other	2 (11.1)	1 (5.9)	
Other	6 (42.9)	2 (16.7)	0.161				

Excellent school performance is defined as average grades of 9 or above of 10. Social media is Facebook, Twitter, and Zalo. Groups were balanced for all variables but household size before the start of the study.

1 5% significance.

### Adherence, acceptability, and usability of FRANI

**FIGURE 5:**
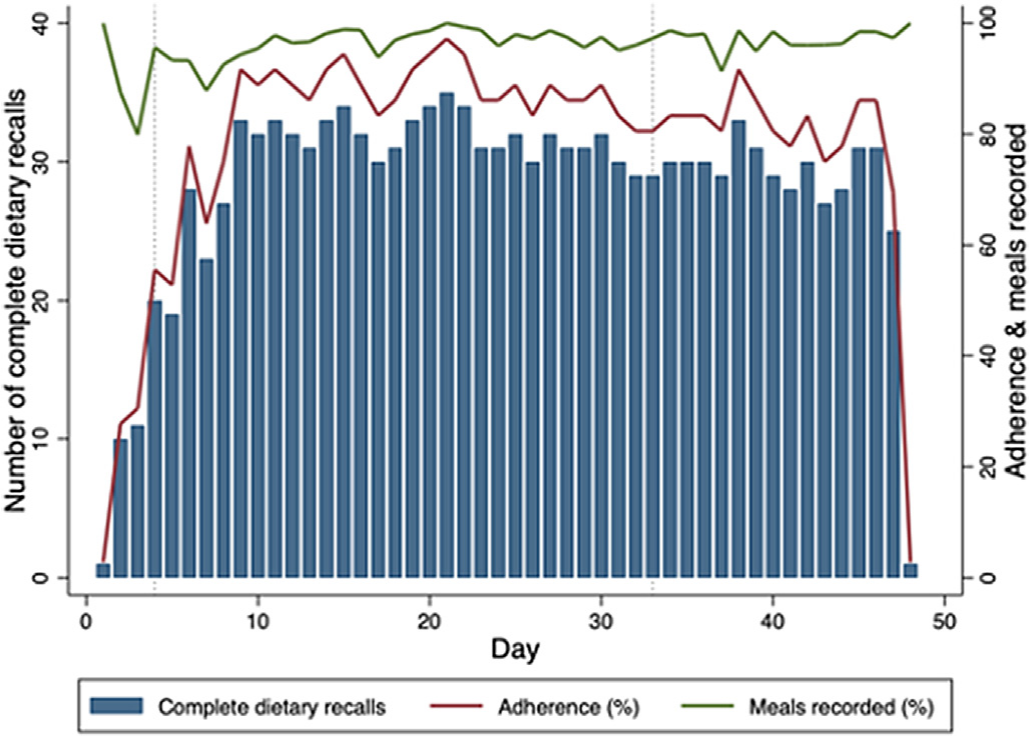
Number of complete food records, adherence (percentage of complete food records), and percentage of meals recorded per day. The first vertical bar from left to right marks the start of the randomized controlled pilot period, and the second marks the end of the randomized pilot and the start of the feasibility period.

The feasibility study period included 412 completed food records over 504 participant-days, an adherence of 82%. The percentage of meals recorded was 97%. Of the 36 participants, 35 deemed FRANI as acceptable and 35 deemed FRANI as usable. Acceptability and usability questions and answers are summarized in Table 2. We measured the number of completed food record by group and period as an exploratory analysis. During the randomized pilot period, the control group completed 473 food record and the intervention group completed 439 of the 540 participant-days per group, with an adherence of 88% and 81%, respectively. During the whole study period, there were 1304 complete food records over 1548 participant-days (36 participants and 43 d), an adherence of 84%. The number of complete food records per day, adherence, and percentage of meals recorded are illustrated in Figure 5.

**TABLE 2:** Agreement with acceptability and usability statements about FRANI^1^

	Strongly agree	Agree	Neither	Disagree	Strongly disagree
Acceptability statements, *n* (%)					
I like this application	8 (22.2)	24 (66.7)	4 (11.1)	0 (0)	0 (0)
I want to keep using this application	2 (5.6)	21 (58.3)	11 (30.6)	2 (5.6)	0 (0)
This application helps me to eat healthy	6 (16.7)	23 (63.9)	4 (11.1)	3 (8.3)	0 (0)
I like receiving badges	9 (25)	24 (66.7)	3 (8.3)	0 (0)	0 (0)
I like the daily diet reports	4 (11.1)	28 (77.8)	4 (11.1)	0 (0)	0 (0)
I like the daily statistics page	7 (19.4)	21 (58.3)	6 (16.7)	2 (5.6)	0 (0)
I like the weekly statistics page	7 (19.4)	22 (61.1)	6 (16.7)	1 (2.8)	0 (0)
I like following other users	2 (5.6)	21 (58.3)	10 (27.8)	3 (8.3)	0 (0)
I like to be part of a team	1 (2.8)	25 (69.4)	10 (27.8)	0 (0)	0 (0)
I like the general navigation of the screens	6 (16.7)	20 (55.6)	9 (25)	1 (2.8)	0 (0)
Usability statements, n (%)					
This application is simple to understand	15 (41.7)	17 (47.2)	2 (5.6)	2 (5.6)	0 (0)
I felt comfortable using this application	7 (19.4)	19 (52.8)	9 (25)	1 (2.8)	0 (0)
I felt praised when I ate healthy foods	13 (36.1)	18 (50)	5 (13.9)	0 (0)	0 (0)
I felt supported by my application friends	4 (11.1)	18 (50)	11 (30.6)	3 (8.3)	0 (0)
I can take pictures of my meals at home	10 (27.8)	24 (66.7)	2 (5.6)	0 (0)	0 (0)
I can take pictures of my meals in school	6 (16.7)	19 (52.8)	8 (22.2)	3 (8.3)	0 (0)
My family lets me take pictures of my meals	10 (27.8)	25 (69.4)	1 (2.8)	0 (0)	0 (0)
I can separate only what I eat to take pictures	6 (16.7)	20 (55.6)	6 (16.7)	4 (11.1)	0 (0)
I trust this application’s information about healthy eating	12 (33.3)	21 (58.3)	3 (8.3)	0 (0)	0 (0)
I trust this application’s feedback about my diet	14 (38.9)	21 (58.3)	1 (2.8)	0 (0)	0 (0)

1 Likert answers are in number (percentage) of participants.

### Food consumption trends

**FIGURE 6:**
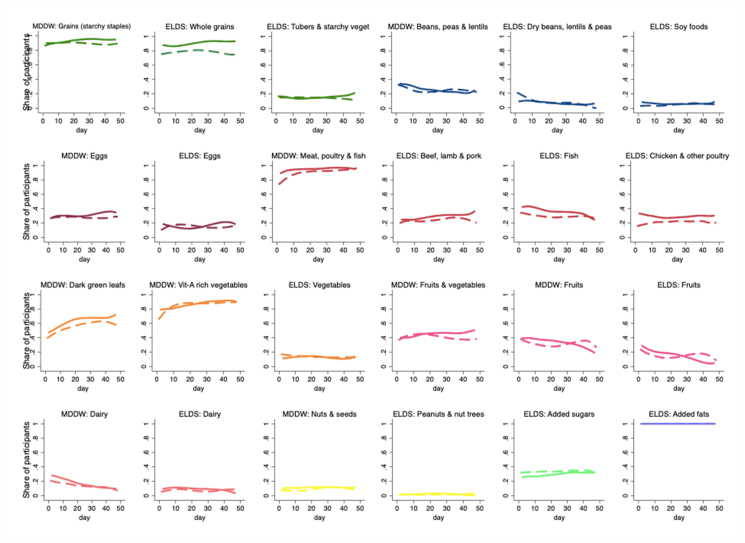
Share of participants who consumed each of the food groups of the MDDW and the ELDS per day during the whole study period. Solid lines are for the intervention group and dashed for the control group. Colors divide types of food groups. ELDS, Eat-Lancet Diet Score; MDDW, Minimum Dietary Diversity for Women.

The randomized pilot period included 995 participant-day food records. When examining consumption of the MDDW food groups, 92% of the participant-days showed consumption of grains, 93% meat and fish, and 87% other vitamin A–rich vegetables, but only 10% nuts, 17% dairy, 26% pulses, and 30% eggs. According to the ELDS, all participant-days showed consumption of a healthy amount of fat, 64% a healthy amount of fish, beef, or poultry, but only 2% a healthy amount of nuts, 8% of beans, 14% of vegetables, 15% of fruits, and 16% of eggs (Figure 6). The majority of the sample did not consume most food groups of the MDDW or ELDS for most days, but there was also a high interday variability.

### Effects of FRANI on food consumption

Participants in the intervention group (gamified FRANI) had higher MDDW (*B*: 1.71 points; 95% CI: 0.98, 1.18; *P* = 0.131) and ELDS (*B*: 1.09 points; 95% CI: 1.01, 1.18; *P* = 0.032) than participants in the control group during the randomized pilot period. There was no evidence of an interaction between intervention status and time (Table 3). Including household size in the regressions to account for differences at baseline between intervention and control groups did not affect the results. Detailed analyses on food group consumption suggested that participants in the intervention group had higher odds of consuming all MDDW food groups than participants in the control group, although the effects were not significant. Participants in the intervention group had higher odds of consuming 8 of the 14 food group of the ELDS within the healthy interval than participants in the control group (Supplemental Table 2). The effects of the intervention on the food group scores for rice, wheat, corn, and other (*B*: 2.36; 95% CI: 1.13, 4.94; *P* = 0.023) and fish (*B*: 1.51; 95% CI: 0.99, 2.29; *P* = 0.054) were statistically significant. The exploratory analysis showed that being in the intervention group did not change the GDQS (*B*: 1.02 points; 95% CI: 0.96, 1.08; *P* = 0.595) compared with the control group. The GDQS food groups with the highest odds of consumption lead by gamified FRANI compared with those by control FRANI were whole grains (*B*: 5.10; 95% CI: 0.55, 47.68; *P* = 0.154), followed by white roots and tubers (*B*: 3.23; 95% CI: 0.54, 19.21; *P* = 0.198), and low-fat dairy (*B*: 2.9; 95% CI: 0.21, 2.13; *P* = 0.430). The GDQS with the lowest odds was refined grains and baked goods (*B*: 0.48; 95% CI: 0.16, 1.47; *P* = 0.199). The effect of intervention on the GDQS and its food groups are in Supplemental Tables 3 and 4a–4c. None of the GDQS results were statistically significant.

**TABLE 3:** Impact of the intervention, time, and the interaction of intervention and time on the Minimum Dietary Diversity for Women and the Eat-Lancet Diet Score.

	MDDW (1)	MDDW (2)	ELDS (3)	ELDS (4)
Intervention	1.07	1.07	1.09	1.07
CI	0.98, 1.18	0.92, 1.26	1.01, 1.18	0.91, 1.26
*P*	0.131	0.371	0.032^1^	0.41
Time		1.00		1.00
CI		0.98, 1.01		1.00, 1.01
*P*		0.52		0.78
Intervention-time		1.00		1.00
CI		0.99, 1.01		0.99, 1.01
*P*		0.995		0.785
Constant	4.68	4.54	3.67	3.62
Observations (n)	995	995	995	995

The maximum score for the MDDW was 10 and for the ELDS was 14, Results of the Poisson models with incidence rate ratios and 36 clusters, 1 for each participant. The intervention group used gamified FRANI. Time is the effect of using FRANI throughout the randomized study period. Models 1 and 3 had intervention as independent variable; models 2 and 4 also included time and the interaction of intervention and time.

ELDS, Eat-Lancet Diet Score; MDDW, Minimum Dietary Diversity for Women.

1 5% significance.

## Discussion

Evidence generated in this study suggests that FRANI, previously validated for dietary assessment with female adolescents in Vietnam and Ghana, is feasible for use in this population. Adherence was high, although a slight downward trend was observed at the end of the study period. Previous research have shown that recording dietary intake with mobile telephones over longer periods is possible, but it is not yet clear what features foster long-term use [14]. Qualitative work showed that using FRANI in school and at home could be challenging because telephones were discouraged in the former and the separation of individual food portions from shared meals was considered disrespectful to family members in Vietnam [20]. However, our study showed that the majority of participants were able to take the pictures at home and school, indicating that the teachers and family supported the use of FRANI. Gamified and control FRANI had a slider feature to indicate how much of the food from the picture the participant ate so to digitally separate the part they consumed from the rest of the shared meal (Figure 3). There was high acceptability and usability, and the only 2 participants that did not consider FRANI acceptable or usable were from the control group. The results also showed that participants trusted the information on healthy eating and feedback on their diet provided by FRANI. Adolescents are particularly sensitive and selective about information to finally make their own decisions and form lifelong habits [47]. This facilitates food promotion of various sources, from science-based applications to pervasive industry marketing [[48], [49], [50]].

Evidence from this randomized pilot suggests that the use of gamified FRANI was associated with a higher dietary quality of female adolescents. FRANI increased the MDDW and the ELDS scores, although the impact on MDDW score was not significant. Participants may have followed the gamified nudges to achieve the goals related to the ELDS so to have more sustainable diets [51]. An exploratory analysis indicated that improvement in dietary quality in the intervention group was driven by consumption of micronutrient-rich foods. Researchers and policymakers interpret increases in the number of different food groups consumed daily from the MDDW and ELDS as something positive for female’s health. There is a high risk of malnutrition if MDDW is ≤5. The average number of MDDW food groups was 4.68 in the control group but increased 1.07 points with the use of gamified FRANI. Our results suggested that gamified FRANI have the potential to increase dietary quality, therefore decrease the risk of malnutrition. However, the group comparison focused on dietary quality was likely underpowered because it was a secondary outcome. We expect to analyze a larger, powered study with dietary quality and malnutrition as primary outcomes to appropriately test the effect of FRANI in dietary quality and risk of malnutrition.

Before the use of FRANI, participants did not know what to make of nutrition and health messages [19,46]. The literature shows that effective communication on nutrition literacy does not happen by pushing messages to people [[52], [53], [54], [55]]. Acting on information requires comprehension, and the most effective way of learning is through experience [56]. The impact of FRANI in behavior change may be attributed to experiential learning. The game elements worked as practical instead of theoretical knowledge in the form of badges and statistics. The knowledge from this feedback could be incorporated to the food choices of the next meal so to achieve new diet-related goals.

FRANI social media features such as sharing badges with friends and receiving positive feedback-only from peers were well-received, corroborating previous qualitative findings [20]. Adolescents have a deep need for social connection, exploration, and autonomous identity [57]. To avoid cyberbullying, FRANI was designed to not allow any written comments in the activity feed because adolescents are easily influenced by peers [58,59]. Participants felt praised and supported by their peers using FRANI team activities. It is possible that gamification sustained behavior change by activating the desired social norms when participants competed and cooperated to achieve team-based goals [30,60]. Gamification may have also allowed self-efficacy by peer modeling, the observation that one can master an activity if others are mastering it too [61]. The 2-d troubleshooting period before the start of the trial resolved problems related to the use of FRANI, an advantage of these kind of data collection method. Real-time data can be used to detect and address adherence issues and procedural problems early on, whereas real-time communication can be used to request information from participants, fix incorrect entries, and give personalized dietary advice in intervention studies [14].

The main strengths of this study are the randomized design using an application developed according to input of potential users that was previously validated for dietary assessment in the population of interest [20,21]. This resulted in high adherence throughout the pilot period, which in turn led to an unprecedented data set with 6 full weeks of food consumption records. The success of data collection may be in part attributed to the teachers and research personnel, which prompted the participants in-person weekly to collect data. This is a limitation for our adherence results because in-person motivation is not replicable in real-life, practical settings. However, the in-person sessions helped to show that the application itself is perfectly functional and that the answers for usability and acceptability are reliable given that participants actually used the application. None of the measures used for dietary quality was perfect for adolescent health. One of the problems of having a sample from a neglected population such as adolescents is that no measures have been developed exclusively for them. An important limitation of the study from the internal validity perspective is its small sample size, which may explain the insignificance of the impact of gamified FRANI on the MDDW. In addition, the study sample was randomly drawn from a population of adolescents enrolled in a high school and, thus, has limited external validity. To our knowledge, this is the first study to assess the feasibility and impact of using an AI-assisted smartphone application to improve the dietary quality of female adolescents in low-income and middle-income countries [20,21]. The next steps of this project are to assess the potential of FRANI to have an impact at scale in different contexts and do a cost-effectiveness analysis.

## Author contributions

The authors’ responsibilities were as follows – AG, BCB, GP, NK: designed the study; AG, BCB, DTTT, LMT, NTH, PHN, THTT: conducted research; provided essential materials for research; BCB, BB, FD, GF, RG, BK, PM, DH, AG: analyzed the data; BCB, AG: wrote the first draft of the manuscript; BCB, AG, PHN: had primary responsibility for the final content; and all authors: read and approved the final manuscript.

## Conflict of interest

PHN is a member of the *Journal of Nutrition* editorial board. All other authors report no conflicts of interest.

## Funding

This research was supported by a Foundation Botnar grant (REG 19-018) and by the CGIAR program on Agriculture, Nutrition and Health (A4NH) (https://www.cgiar.org/research/program-platform/agriculture-nutrition-health/) BCB was a PhD candidate supported by the Friedman Nutrition and Citizenship Fellowship from the Friedman School of Nutrition Science and Policy at Tufts University.

## Data availability

Data described in this manuscript, code book, and analytic code will be made available on request pending application and approval.

## Appendix A Supplementary data

Multimedia component1 Multimedia component1

## References

[1] Norris S.A., Frongillo E.A., Black M.M., Dong Y., Fall C., Lampl M. (2022). Nutrition in adolescent growth and development. Lancet.

[2] Falconi A., Gemmill A., Dahl R.E., Catalano R. (2014). Adolescent experience predicts longevity: evidence from historical epidemiology. J. Dev. Orig Health Dis..

[3] Depauw E., Oxley D. (2019). Toddlers, teenagers, and terminal heights: the importance of puberty for male adult stature, Flanders, 1800–76. Econ. Hist. Rev..

[4] Spear B.A. (2002). Adolescent growth and development. J. Am. Diet. Assoc..

[5] Diethelm K., Jankovic N., Moreno L.A., Huybrechts I., Henauw S.D., Vriendt T. (2012). Food intake of European adolescents in the light of different food-based dietary guidelines: results of the HELENA (Healthy Lifestyle in Europe by Nutrition in Adolescence) study. Public Health Nutr.

[6] Cunha C.M., Costa P.R.F., de Oliveira L.P.M., Queiroz V.A.O., Pitangueira J.C.D., Oliveira A.M. (2018). Dietary patterns and cardiometabolic risk factors among adolescents: systematic review and meta-analysis. Br. J. Nutr..

[7] Dahm C.C., Chomistek A.K., Jakobsen M.U., Mukamal K.J., Eliassen A.H., Sesso H.D. (2016). Adolescent diet quality and cardiovascular disease risk factors and incident cardiovascular disease in middle-aged women. J. Am. Heart Assoc..

[8] Patton G.C., Sawyer S.M., Santelli J.S., Ross D.A., Afifi R., Allen N.B. (2016). Our future: a Lancet commission on adolescent health and wellbeing. Lancet.

[9] Hargreaves D., Mates E., Menon P., Alderman H., Devakumar D., Fawzi W. (2022). Strategies and interventions for healthy adolescent growth, nutrition, and development. Lancet.

[10] Bell W., Colaiezzi B.A., Prata C.S., Coates J.C. (2017). Scaling up dietary data for decision-making in low-income countries: new technological frontiers. Adv. Nutr..

[11] Rangan A.M., Tieleman L., Louie J.C.Y., Tang L.M., Hebden L., Roy R. (2016). Electronic dietary intake assessment (e-DIA): relative validity of a mobile phone application to measure intake of food groups. Br. J. Nutr..

[12] Carter M.C., Burley V.J., Nykjaer C., Cade J.E. (2013). ‘My Meal Mate’ (MMM): validation of the diet measures captured on a smartphone application to facilitate weight loss. Br. J. Nutr..

[13] Rollo M.E., Ash S., Lyons-Wall P., Russell A. (2011). Trial of a mobile phone method for recording dietary intake in adults with type 2 diabetes: evaluation and implications for future applications. J. Telemed. Telecare..

[14] Sharp D.B., Allman-Farinelli M. (2014). Feasibility and validity of mobile phones to assess dietary intake. Nutrition.

[15] Bromage S., Batis C., Bhupathiraju S.N., Fawzi W.W., Fung T.T., Li Y. (2021). Development and validation of a novel food-based global diet quality score (GDQS). J. Nutr..

[16] Anderson M., Jiang J. (2018). https://www.pewresearch.org/internet/2018/05/31/teens-social-media-technology-2018/.

[17] Lenhart A. (2015). https://www.pewresearch.org/internet/2015/04/09/teens-social-media-technology-2015/.

[18] Rangan A.M., O’Connor S., Giannelli V., Yap M.L., Tang L.M., Roy R. (2015). Electronic dietary intake assessment (e-DIA): comparison of a mobile phone digital entry app for dietary data collection with 24-hour dietary recalls. JMIR MHealth UHealth.

[19] Braga B.C.C., Aberman N.L., Arrieta A., Bannerman B., Burns A., Folson G. (2021).

[20] Braga B.C., Nguyen P.H., Aberman N.-L., Doyle F., Folson G., Hoang N. (2022). Exploring an artificial intelligence–based, gamified phone app prototype to track and improve food choices of adolescent girls in Vietnam: acceptability, usability, and likeability study. JMIR Form. Res..

[21] Nguyen P.H., Tran L.M., Hoang N.T., Trương D.T.T., Tran T.H.T., Huynh P.N. (2022). Relative validity of a mobile AI-technology–assisted dietary assessment in adolescent females in Vietnam. Am. J. Clin. Nutr..

[22] Nguyen P.H., Tran L.M., Hoang N.T., Trương D.T., Tran T.H., Huynh P.N. (2021). https://ideas.repec.org/b/fpr/ifprib/1290144001.html.

[23] Folson G.K., Bannerman B., Atadze V., Ador G., Kolt B., McCloskey P. (2023). Validation of mobile artificial intelligence technology–assisted dietary assessment tool against weighed records and 24-hour recall in adolescent females in Ghana. J. Nutr..

[24] Gloria F., Boateng B., Gabriel A., Vicentia A., Saudatu A., Stephen A. (2022).

[25] Ezezika O., Oh J., Edeagu N., Boyo W. (2018). Gamification of nutrition: a preliminary study on the impact of gamification on nutrition knowledge, attitude, and behaviour of adolescents in Nigeria. Nutr. Health.

[26] Maher C.A., Lewis L.K., Ferrar K., Marshall S., Bourdeaudhuij I.D., Vandelanotte C. (2014). Are health behavior change interventions that use online social networks effective? A systematic review. J. Med. Internet Res..

[27] Laranjo L., Arguel A., Neves A.L., Gallagher A.M., Kaplan R., Mortimer N. (2015). The influence of social networking sites on health behavior change: a systematic review and meta-analysis. J. Am. Med Inform. Assoc..

[28] Bort-Roig J., Gilson N.D., Puig-Ribera A., Contreras R.S., Trost S.G. (2014). Measuring and influencing physical activity with smartphone technology: a systematic review. Sports Med.

[29] Baranowski T., Ryan C., Hoyos-Cespedes A., Lu A.S. (2019). Nutrition education and dietary behavior change games: a scoping review. Games Health J.

[30] Azevedo J., Padrão P., Gregório M.J., Almeida C., Moutinho N., Lien N. (2019). A web-based gamification program to improve nutrition literacy in families of 3- to 5-year-old children: the Nutriscience project. J. Nutr. Educ. Behav..

[31] Yoshida-Montezuma Y., Ahmed M., Ezezika O. (2020). Does gamification improve fruit and vegetable intake in adolescents? A systematic review. Nutr. Health.

[32] Suleiman-Martos N., García-Lara R.A., Martos-Cabrera M.B., Albendín-García L., Romero-Béjar J.L., Cañadas-De la Fuente G.A. (2021). Gamification for the improvement of diet, nutritional habits, and body composition in children and adolescents: a systematic review and meta-analysis. Nutrients.

[33] Braga B.C., Cash S.B., Sarson K., Chang R., Mosca A., Wilson N.L.W. (2023). The gamification of nutrition labels to encourage healthier food selection in online grocery shopping: a randomized controlled trial. Appetite.

[34] Braga B.C., Cash S.B., Sarson K., Chang R., Mosca A., Wilson N.L.W. (2023). The creation of an online grocery store for experimental purposes: a pilot study. Food Qual. Prefer..

[35] Gan K.O., Allman-Farinelli M. (2011). A scientific audit of smartphone applications for the management of obesity. Aust. N. Z. J. Public Health..

[36] Food and Agriculture Organization of the United Nations (2022). http://www.fao.org/nutrition/education/food-dietary-guidelines/regions/vietnam/en/.

[37] United States Department of Agriculture, Washington, DC (2022). https://www.myplate.gov/eat-healthy/what-is-myplate.

[38] Arimond M., Wiesmann D., Becquey E., Carriquiry A., Daniels M.C., Deitchler M. (2010). Simple food group diversity indicators predict micronutrient adequacy of women’s diets in 5 diverse, resource-poor settings. J. Nutr..

[39] Food and Agricultural Organization (2021). https://www.fao.org/3/cb3434en/cb3434en.pdf.

[40] Women’s Dietary Diversity Project (WDDP) Study Group (2017). Development of a dichotomous indicator for population-level assessment of dietary diversity in women of reproductive age. Curr. Dev. Nutr..

[41] Knuppel A., Papier K., Key T.J., Travis R.C. (2019). EAT-Lancet score and major health outcomes: the EPIC-Oxford study. Lancet.

[42] Hanley-Cook G.T., Argaw A.A., de Kok B.P., Vanslambrouck K.W., Toe L.C., Kolsteren P.W. (2021). EAT–Lancet diet score requires minimum intake values to predict higher micronutrient adequacy of diets in rural women of reproductive age from five low- and middle-income countries. Br. J. Nutr..

[43] Willett W., Rockström J., Loken B., Springmann M., Lang T., Vermeulen S. (2019). Food in the Anthropocene: the EAT–Lancet Commission on healthy diets from sustainable food systems. Lancet.

[44] Nhà X. BẢNG THÀNH PHẦN THỰC PHẨM VIỆT NAM. https://www.fao.org/fileadmin/templates/food_composition/documents/pdf/VTN_FCT_2007.pdf.

[45] Allam A., Kostova Z., Nakamoto K., Schulz P.J. (2015). The effect of social support features and gamification on a web-based intervention for rheumatoid arthritis patients: randomized controlled trial. J. Med Internet Res..

[46] Braga B.C., Arrieta A., Bannerman B., Doyle F., Folson G., Gangupantulu R. (2022). Measuring adherence, acceptability and likability of an artificial-intelligence-based, gamified phone application to improve the quality of dietary choices of adolescents in Ghana and Vietnam: protocol of a randomized controlled pilot test. Front. Digit. Health.

[47] Neufeld L.M., Andrade E.B., Ballonoff Suleiman A., Barker M., Beal T., Blum L.S. (2022). Food choice in transition: adolescent autonomy, agency, and the food environment. Lancet.

[48] Aboujaoude E., Savage M.W., Starcevic V., Salame W.O. (2015). Cyberbullying: review of an old problem gone viral. J. Adolesc. Health.

[49] O’Keeffe G.S., Clarke-Pearson K. (2011). Council on Communications and Media, The impact of social media on children, adolescents, and families. Pediatrics.

[50] Starcevic V., Aboujaoude E. (2015). Cyberchondria, cyberbullying, cybersuicide, cybersex: “new” psychopathologies for the 21st century?. World Psychiatry.

[51] Crandon T.J., Scott J.G., Charlson F.J., Thomas H.J. (2022). A social–ecological perspective on climate anxiety in children and adolescents. Nat. Clim Change..

[52] Cullen T., Hatch J., Martin W., Higgins J.W., Sheppard R. (2015). Food literacy: definition and framework for action. Can. J. Diet. Pract. Res..

[53] Perry E.A., Thomas H., Samra H.R., Edmonstone S., Davidson L., Faulkner A. (2017). Identifying attributes of food literacy: a scoping review. Public Health Nutr.

[54] Truman E., Lane D., Elliott C. (2017). Defining food literacy: a scoping review. Appetite.

[55] Velardo S. (2015). The nuances of health literacy, nutrition literacy, and food literacy. J. Nutr. Educ. Behav..

[56] Dudley D.A., Cotton W.G., Peralta L.R. (2015). Teaching approaches and strategies that promote healthy eating in primary school children: a systematic review and meta-analysis. Int. J. Behav. Nutr. Phys. Act..

[57] Montgomery K.C., Chester J., Grier S.A., Dorfman L. (2012). The new threat of digital marketing. Pediatr. Clin. North. Am..

[58] Tremblay L., Lariviere M. (2009). The influence of puberty onset, body mass index, and pressure to be thin on disordered eating behaviors in children and adolescents. Eat. Behav..

[59] Contento I.R., Williams S.S., Michela J.L., Franklin A.B. (2006). Understanding the food choice process of adolescents in the context of family and friends. J. Adolesc. Health.

[60] Higgs S., Ruddock H., Meiselman H.L. (2020). Handbook of Eating and Drinking: Interdisciplinary Perspectives.

[61] Bandura A. (2022). https://scholar.google.com/scholar_lookup?titleSocial20foundations20for20thought20and20action3A20A20social20cognitive20theory&authorA.20Bandura&publication_year1986.

