# Supplementary material for: Feasibility of Using an Artificial Intelligence-based Telephone Application for Dietary Assessment and Nudging to Improve the Quality of Food Choices of Female Adolescents in Vietnam: Evidence from a Randomized Pilot Study
Source: Curr Dev Nutr. 2023 Dec 13;8(6):102063. doi: 10.1016/j.cdnut.2023.102063 (PMC11137395; doi:10.1016/j.cdnut.2023.102063)
Supplement: Multimedia component1 [file mmc1.docx]

| Supplementary Table 1: Characteristics of FRANI by level of functionality | | |
| --- | --- | --- |
|  | Treatment  Gamified | Control  Limited |
| Account registration and login | Available | Available |
| Home dashboard | Available | N/A |
| Setting goals | Available | N/A |
| Meal entry (picture taking) | Available | Available |
| Scores and statistics | Available | N/A |
| Medals and badges | Available | N/A |
| Daily report | Available | N/A |
| Activity feed | Available | Modified |
| Notification | Available + Daily report reminder | Available |
| Note: N/A stands for not available. The daily report was available at the end of each day on FRANI and was sent as a notification. | | |

A
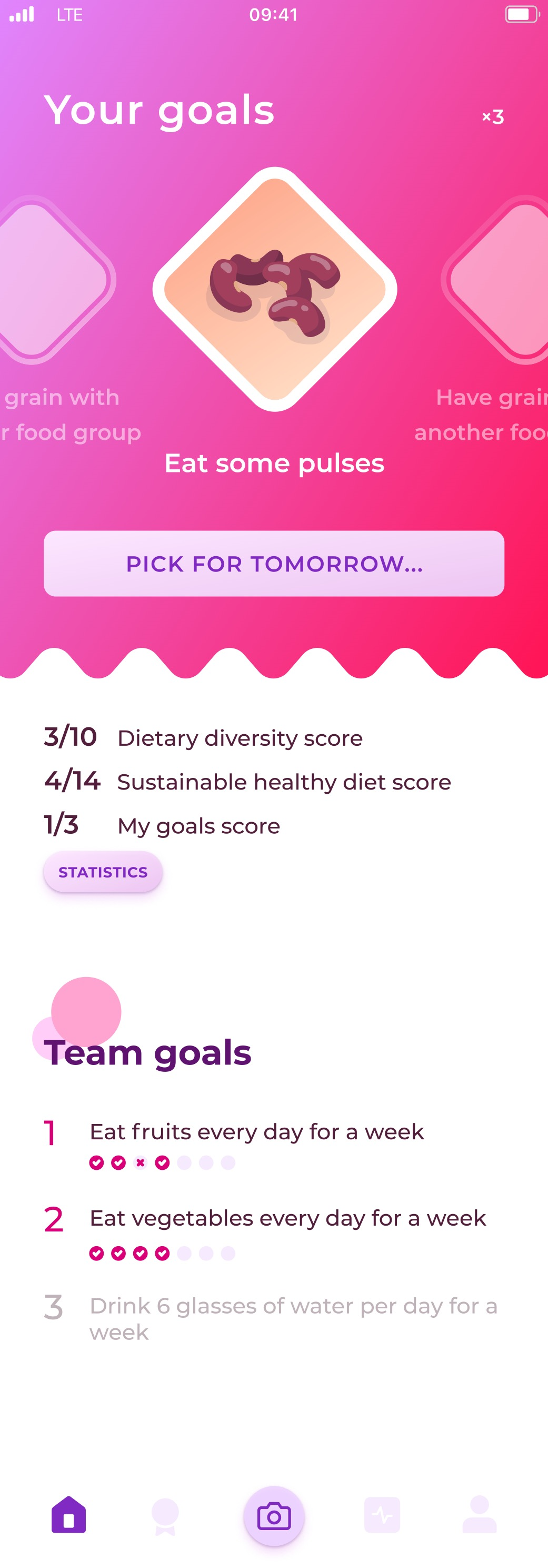
 B
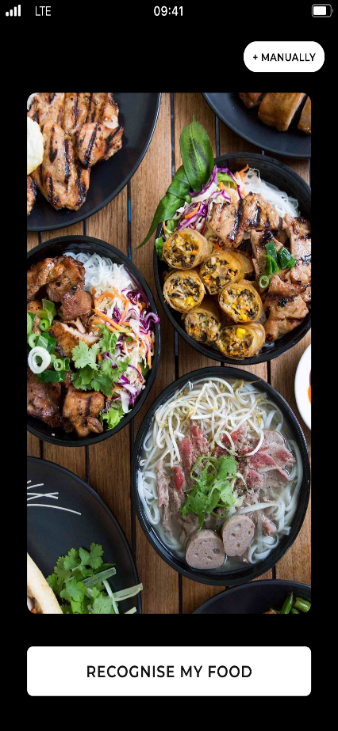
 C
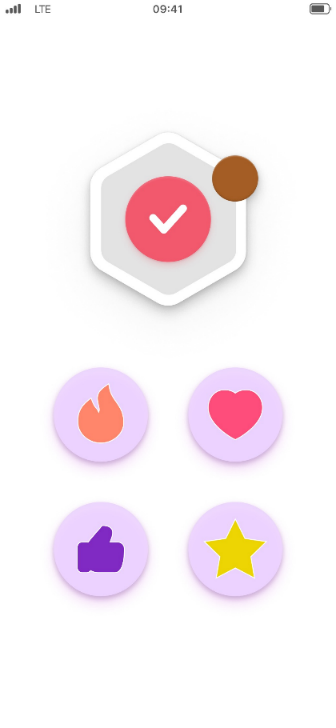


D
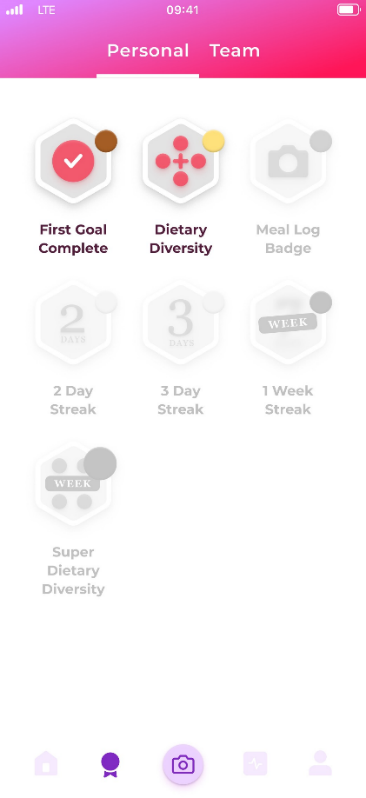
 E
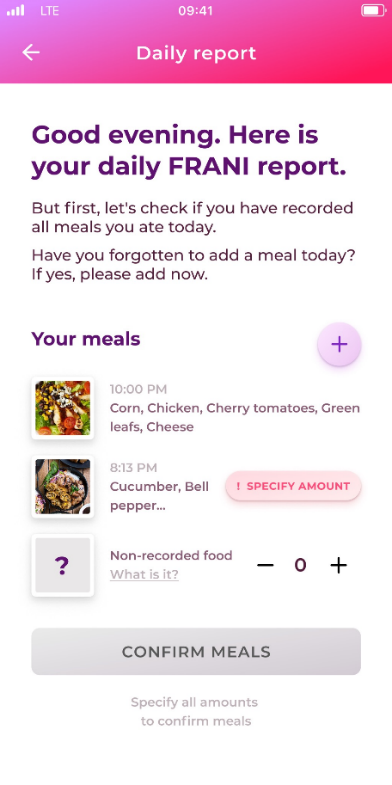


**Supplementary Figure 1:** Wireframe A shows the home dashboard with dietary scores and B is the camera frame. Wireframe C shows the confirmation that the foods were uploaded with a bronze badge and D shows individual-based badges. Wireframe E shows FRANI daily report sent in the evening so users can include, complete or correct information on food consumption uploaded throughout the day.

| **Supplementary Table 2: Impact of the intervention, time and the interaction of intervention and time on each food group of the Eat-Lancet Diet Score** | | | | | | | | | | | | | |
| --- | --- | --- | --- | --- | --- | --- | --- | --- | --- | --- | --- | --- | --- |
|  | Rice, wheat, corn and other | Potatoes and cassava | Dry beans, lentils and peas | Soy foods | Peanuts and tree nuts | Whole milk or derivative equivalents | Beef, lamb, pork | Chicken and other poultry | Fish | Eggs | All vegetables | All fruits | All sweeteners |
| **Bivariable** |  |  |  |  |  |  |  |  |  |  |  |  |  |
| Intervention | 2.36 | 0.95 | 0.95 | 1.63 | 1.80 | 1.60 | 1.27 | 1.53 | 1.51 | 0.80 | 0.97 | 1.53 | 0.9 |
| CI | 1.13; 4.94 | 0.52; 1.73 | 0.45; 2.01 | 0.68; 3.88 | 0.43; 7.54 | 0.71; 3.58 | 0.92; 1.76 | 0.94; 2.51 | 0.99; 2.29 | 0.46; 1.40 | 0.33; 2.86 | 0.67; 3.50 | 0.55; 1.30 |
| P-value | 0.023** | 0.869 | 0.899 | 0.273 | 0.421 | 0.255 | 0.147 | 0.087 | 0.054** | 0.438 | 0.949 | 0.316 | 0.451 |
| Constant | 5.04 | 0.16 | 0.06 | 0.03 | 0.01 | 0.05 | 0.32 | 0.27 | 0.40 | 0.18 | 0.09 | 0.00 | 0.49 |
| **Multivariable** |  |  |  |  |  |  |  |  |  |  |  |  |  |
| Intervention | 1.13 | 0.80 | 0.98 | 4.09 | 1.448 | 1.25 | 1.18 | 1.81 | 1.92 | 0.64 | 0.64 | 2.59 | 0.56 |
| CI | 0.38; 3.34 | 0.29; 2.18 | 0.29; 3.37 | 0.76; 22.00 | 0.10; 21.29 | 0.36; 4.36 | 0.57; 2.45 | 0.79; 4.14 | 0.92; 4.02 | 0.25; 1.67 | 0.16; 2.62 | 0.74; 9.10 | 0.26; 1.19 |
| P-value | 0.831 | 0.663 | 0.974 | 0.10 | 0.787 | 0.73 | 0.66 | 0.161 | 0.61 | 0.363 | 0.536 | 0.137 | 0.132 |
| Time | 1.02 | 1.00 | 0.97 | 1.06 | 1.03 | 0.96 | 1.02 | 1.01 | 0.99 | 0.99 | 0.99 | 1.03 | 1.00 |
| CI | 0.99; 1.05 | 0.97; 1.03 | 0.93; 1.01 | 1.00; 1.11 | 0.94; 1.120 | 0.92; 1.00 | 0.99; 1.04 | 0.99; 1.04 | 0.97; 1.02 | 0.96; 1.02 | 0.95; 1.02 | 0.99; 1.07 | 0.98; 1.02 |
| P-value | 0.264 | 0.889 | 0.124 | 0.052** | 0.535 | 0.076 | 0.171 | 0.389 | 0.608 | 0.397 | 0.449 | 0.132 | 0.877 |
| Intervention-time | 1.05 | 1.01 | 1.00 | 0.96 | 1.011 | 1.01 | 1.00 | 0.99 | 0.99 | 1.01 | 1.02 | 0.97 | 1.02 |
| CI | 1.00; 1.10 | 0.97; 1.05 | 0.94; 1.06 | 0.89; 1.02 | 0.907; 1.127 | 0.96; 1.07 | 0.97; 1.04 | 0.96; 1.03 | 0.96; 1.02 | 0.97; 1.06 | 0.97; 1.07 | 0.93; 1.02 | 0.99; 1.06 |
| P-value | 0.064 | 0.673 | 0.935 | 0.205 | 0.844 | 0.625 | 0.803 | 0.64 | 0.421 | 0.577 | 0.371 | 0.271 | 0.186 |
| Constant | 3.74 | 0.16 | 0.11 | 0.01 | 0.004 | 0.11 | 0.23 | 0.22 | 0.44 | 0.22 | 0.11 | 0.05 | 0.50 |
| Note: Results of Logistic models with 36 clusters and 995 participant-days for the all food groups that compose the Eat-Lancet Diet Score (ELDS) but fat since the outcome did not vary for this food group. CI=confidence interval. **1% significance; *5% significance. The intervention group used gamified FRANI. Bivariable models have intervention as independent variable; multivariable models also include time, and the interaction of intervention and time. | | | | | | | | | | | | | |

| **Supplementary Table 3: Impact of the intervention, time and the interaction of intervention and time on the Global Diet Quality Score.** | | | | | | | |
| --- | --- | --- | --- | --- | --- | --- | --- |
|  | GDQS (1) | GDQS (2) | GDQS+ (3) | GDQS+ (4) | GDQS- (5) | GDQS- (6) |  |
| Intervention | 1.02 | 1.00 | 1.04 | 1.03 | 1.00 | 0.98 |  |
| CI | [0.96; 1.08] | [0.91; 1.09] | [0.82; 1.33] | [0.78; 1.35] | [0.96; 1.04] | [0.90; 1.07] |  |
| P-value | 0.595 | 0.968 | 0.744 | 0.858 | 0.868 | 0.647 |  |
| Time |  | 1.00 |  | 1.00 |  | 1.00 |  |
| CI |  | [1.00; 1.00] |  | [1.00; 1.00] |  | [1.00; 1.00] |  |
| P-value |  | 0.600 |  | 0.956 |  | 0.559 |  |
| Intervention-time |  | 1.00 |  | 1.00 |  | 1.00 |  |
| CI |  | [1.00; 1.01] |  | [0.99; 1.01] |  | [1.00; 1.01] |  |
| P-value |  | 0.587 |  | 0.804 |  | 0.672 |  |
| Constant | 17.21 | 17.43 | 4.61 | 4.62 | 12.42 | 12.63 |  |
| Observations | n=992 | n=992 | n=992 | n=992 | n=992 | n=992 |  |
| Note: GDQS: Global Diet Quality Score. CI=confidence interval. Results of the Poisson models with incidence rate ratios and 36 clusters, one for each participant. The intervention group used the gamified FRANI. Time is the effect of using FRANI throughout the study period. All models have intervention as independent variable; models 2, 4 and 6 also include time, and the interaction of intervention and time. The GDQS+ is the total score of the healthy food groups with a possible range from 0 to 32, while the GDQS- is the total score of the unhealthy food groups, with a range between 0 to 17 (Bromage, 2021). | | | | | | |  |

| **Supplementary Table 4a: Impact of the intervention, time and the interaction of intervention and time on each food group of the Global Diet Quality Score** | | | | | | | | |
| --- | --- | --- | --- | --- | --- | --- | --- | --- |
|  | Citrus fruits | Deep orange fruits | Other fruits | Dark green leafy vegetables | Cruciferous vegetables | Deep orange vegetables | Other vegetables | Legumes |
| **Bivariable** |  |  |  |  |  |  |  |  |
| Intervention | 0.99 | 1.97 | 1.35 | 0.96 | 1.08 | 1.30 | 0.73 | 1.07 |
| CI | 0.20; 4.92 | 0.59; 6.57 | 0.58; 3.12 | 0.42; 2.21 | 0.46; 2.53 | 0.68; 2.50 | 0.41; 1.29 | 0.75; 1.54 |
| P-value | 0.988 | 0.270 | 0.490 | 0.920 | 0.862 | 0.428 | 0.280 | 0.710 |
| Constant | 0.01 | 0.02 | 0.17 | 0.70 | 0.11 | 0.10 | 0.73 | 0.27 |
| **Multivariable** |  |  |  |  |  |  |  |  |
| Intervention | 0.12 | 1.25 | 1.66 | 0.70 | 0.79 | 0.51 | 0.78 | 1.15 |
| CI | 0.00; 7.91 | 0.21; 7.50 | 0.54; 5.06 | 0.25; 2.01 | 0.23; 2.70 | 0.17; 1.51 | 0.34; 1.80 | 0.54; 2.45 |
| P-value | 0.316 | 0.805 | 0.376 | 0.508 | 0.709 | 0.220 | 0.557 | 0.717 |
| Time | 0.95 | 0.92 | 1.00 | 1.01 | 1.00 | 0.96 | 1.00 | 0.99 |
| CI | 0.82; 1.09 | 0.86; 0.99 | 0.98; 1.03 | 0.98; 1.03 | 0.97; 1.04 | 0.93; 1.00 | 0.97; 1.02 | 0.96; 1.02 |
| P-value | 0.454 | 0.019** | 0.826 | 0.698 | 0.885 | 0.036** | 0.713 | 0.431 |
| Intervention-time | 1.12 | 1.03 | 0.99 | 1.02 | 1.02 | 1.05 | 1.00 | 1.00 |
| CI | 0.92; 1.37 | 0.95; 1.13 | 0.95; 1.03 | 0.98; 1.05 | 0.97; 1.06 | 1.00; 1.11 | 0.97; 1.03 | 0.96; 1.03 |
| P-value | 0.272 | 0.496 | 0.575 | 0.328 | 0.483 | 0.036** | 0.825 | 0.824 |
| Constant | 0.02 | 0.06 | 0.16 | 0.64 | 0.11 | 0.19 | 0.79 | 0.33 |

| **Supplementary Table 4b: Impact of the intervention, time and the interaction of intervention and time on each food group of the Global Diet Quality Score** | | | | | | | | |
| --- | --- | --- | --- | --- | --- | --- | --- | --- |
|  |  | Nuts and seeds | Whole grains | Liquid oils | Fish and shellfish | Poultry and game meats | Low-fat dairy | Eggs |
| **Bivariable** |  |  |  |  |  |  |  |  |
| Intervention |  | 1.16 | 5.10 | 0.99 | 1.10 | 1.24 | 2.90 | 1.10 |
| CI |  | 0.63; 2.14 | 0.55; 47.68 | 0.56; 1.76 | 0.59; 2.08 | 0.72; 2.13 | 0.21; 40.86 | 0.64; 1.80 |
| P-value |  | 0.644 | 0.154 | 0.967 | 0.763 | 0.440 | 0.430 | 0.699 |
| Constant |  | 0.12 | 0.00 | 1.54 | 0.18 | 0.22 | 0.00 | 0.28 |
| **Multivariable** |  |  |  |  |  |  |  |  |
| Intervention |  | 0.49 | 1.83 | 1.55 | 0.63 | 2.33 | 0.50 | 1.04 |
| CI |  | 0.17; 1.41 | 0.02; 160.41 | 0.67; 3.59 | 0.23; 1.72 | 0.93; 5.84 | 0.00; 81.50 | 0.46; 2.39 |
| P-value |  | 0.187 | 0.791 | 0.306 | 0.370 | 0.072 | 0.791 | 0.919 |
| Time |  | 0.97 | 0.89 | 1.01 | 0.99 | 1.03 | 0.81 | 1.00 |
| CI |  | 0.94; 1.01 | 0.67; 1.19 | 0.99; 1.04 | 0.96; 1.02 | 1.00; 1.02 | 0.54; 1.22 | 0.97; 1.02 |
| P-value |  | 0.111 | 0.438 | 0.337 | 0.615 | 0.023* | 0.310 | 0.701 |
| Intervention-time |  | 1.05 | 1.07 | 0.98 | 1.03 | 0.97 | 1.16 | 1.00 |
| CI |  | 1.00; 1.10 | 0.79; 1.47 | 0.95; 1.01 | 0.99; 1.07 | 0.93; 1.01 | 0.75; 1.80 | 0.97; 1.04 |
| P-value |  | 0.049* | 0.650 | 0.147 | 0.157 | 0.097 | 0.499 | 0.877 |
| Constant |  | 0.20 | 0.01 | 1.24 | 0.21 | 0.12 | 0.01 | 0.31 |
| Note: GDQS=Global Diet Quality Score. CI=confidence interval. **1% significance; *5% significance. Results of Logit models with 36 clusters and 995 participant-days for healthy food groups that compose the Global Diet Quality Score. Deep orange tubers was not reported since the outcome did not vary. The intervention group used gamified FRANI. Univariable models have intervention as independent variable; multivariable models also include time, and the interaction of intervention and time. | | | | | | | | |

| **Supplementary Table 4c: Impact of the intervention, time and the interaction of intervention and time on each food group of the Global Diet Quality Score** | | | | | | | | |  |
| --- | --- | --- | --- | --- | --- | --- | --- | --- | --- |
|  | High-fat dairy | Red meat | Processed meat | Refined grains and baked goods | Sweets and ice creams | Sugar-sweetened beverages | Juice | White roots and tubers | Purchased deep fried foods |
| **Bivariable** |  |  |  |  |  |  |  |  |  |
| Intervention | 1.28 | 1.20 | 1.59 | 0.48 | 1.26 | 1.01 | 1 (omitted) | 3.23 | 1 (omitted) |
| CI | 0.47; 3.47 | 0.70; 2.05 | 0.40; 6.29 | 0.16; 1.47 | 0.34; 4.63 | 0.06; 16.21 | - | 0.54; 19.21 | - |
| P-value | 0.632 | 0.512 | 0.505 | 0.199 | 0.730 | 0.993 | - | 0.198 | - |
| Constant | 0.02 | 1.93 | 53.95 | 0.16 | 90.47 | 491.96 | 245.50 | 108.137 | 163.33 |
| **Multivariable** | |  |  |  |  |  |  |  |  |
| Intervention | 0.96 | 0.58 | 0.20 | 0.74 | 0.09 | 0.11 | 1 (omitted) | 116.00 | 1 (omitted) |
| CI | 0.18; 5.13 | 0.25; 1.31 | 0.02; 1.67 | 0.18; 3.00 | 0.01; 1.15 | 0.00; 200.60 | - | 0.22; 60956.30 | - |
| P-value | 0.964 | 0.189 | 0.136 | 0.675 | 0.064 | 0.568 | - | 0.137 | - |
| Time | 0.95 | 0.99 | 0.95 | 0.99 | 1.00 | 0.94 | 0.93 | 0.94 | 1.02 |
| CI | 0.89; 1.01 | 0.96; 1.01 | 0.90; 1.01 | 0.96; 1.02 | 0.93; 1.09 | 0.73; 1.21 | 0.78; 1.12 | 0.87; 1.02 | 0.89; 1.16 |
| P-value | 0.081 | 0.298 | 0.085 | 0.520 | 0.914 | 0.640 | 0.459 | 0.129 | 0.835 |
| Intervention-time | 1.02 | 1.04 | 1.12 | 0.98 | 1.22 | 1.12 | 1 (omitted) | 0.87 | 1 (omitted) |
| CI | 0.94; 1.11 | 1.01; 1.08 | 1.02; 1.23 | 0.93; 1.02 | 1.02; 1.46 | 0.79; 1.60 | - | 0.700; 1.08 | - |
| P-value | 0.691 | 0.021* | 0.014* | 0.292 | 0.028* | 0.527 | - | 0.211 | - |
| Constant | 0.06 | 2.46 | 151.80 | 0.20 | 94.30 | 1771.92 | 1081.19 | 379.04 | 125.04 |
| Note: Note: CI=confidence interval. GDQS=Global Diet Quality Score. Results of Logit models with 36 clusters and 995 participant-days for the unhealthy food groups of the GDQS. CI=confidence interval. **1% significance; *5% significance. The intervention group used gamified FRANI. Univariable models have intervention as independent variable; multivariable models also include time, and the interaction of intervention and time. | | | | | | | | |  |
